# Antibody Binding Studies Reveal Conformational Flexibility of the *Bacillus cereus* Non-Hemolytic Enterotoxin (Nhe) A-Component

**DOI:** 10.1371/journal.pone.0165135

**Published:** 2016-10-21

**Authors:** A. Didier, R. Dietrich, E. Märtlbauer

**Affiliations:** Department of Veterinary Science, Ludwig-Maximilians Universität München, Oberschleißheim, Germany; US Naval Research Laboratory, UNITED STATES

## Abstract

The non-hemolytic enterotoxin complex (Nhe) is supposed to be the main virulence factor of *B*. *cereus* causing a diarrheal outcome of food poisoning. This tripartite toxin consists of the single components NheA, -B and -C all of them being necessary for maximum toxicity. In the past, research activities aiming to elucidate the mode-of-action of Nhe were mostly focused on the B- and C-component. In this study the generation of novel monoclonal antibodies (mAb) and their thorough characterization enabled the determination of key features for NheA. By the means of immunoaffinity chromatography it could be shown that NheA does not interact with -B and -C in solution. Additionally, the establishment of a highly sensitive sandwich-EIA now enables the detection of NheA in *B*. *cereus* supernatants down to 20 pg ml^-1^.Peptide-based epitope mapping in combination with partially deleted recombinant NheA fragments allowed the allocation of the binding regions for the three mAbs under study. Furthermore, by different EIA set-ups the conformational flexibility of NheA could be shown. For two of the antibodies under study different mechanisms of NheA neutralization were proven. Due to prevention of complete pore formation by one of the antibodies, NheA could be detected in an intermediate stage of the tripartite complex on the cell surface. Taken together, the results obtained in the present study allow a refinement of the mode-of-action for the Nhe toxin-complex.

## Introduction

Spore-forming Gram-positive *Bacillus cereus* strains are capable to induce two types of foodborne disease [1,2]. On the one hand the emetic syndrome is caused by a heat-stable cyclic dodecadepsipeptide named cereulide, which is pre-formed in the food [3]. The diarrheal syndrome on the other hand is related to the presence of either the three component enterotoxin complexes Hbl (hemolysin BL) [4] and Nhe (non-hemolytic enterotoxin) [5] or a single protein CytK-1 (Cytotoxin K-1) [6]. Expression of the diarrhea-associated enterotoxins occurs in the gastrointestinal tract after ingestion of viable bacterial cells or spores [7].

More than 90% of *B*. *cereus* isolates harbor the *nhe* genes and about 50% the *hbl* genes [8,9]. Thus, Nhe tends to be the most important virulence factor responsible for diarrheal disease outcome. Although the aforementioned enterotoxin complex with its constitutive components NheA, NheB and NheC has first been described 15 years ago [10], research on the underlying mode-of-action is still ongoing.

Nhe is supposed to act as a pore forming toxin similar to ClyA—a multimeric toxin expressed in *Enterobacteriaceae* [11]. Nevertheless, one has to keep in mind that ClyA homo-oligomerises upon pore formation [12], whereas the Nhe components will have to form hetero-oligomers. Thus, a more complex mode-of-action has to be assumed.

For a long time, structural data for Nhe components could be only deduced by modeling on the x-ray structures of Hbl-B [13] or ClyA [14]. Meanwhile, the structure of NheA has been resolved [15,16] thus providing novel aspects on the pore-formation process.

Several versatile monoclonal antibodies [17] as well as *in vitro* cell based systems [18,19] have been established and facilitated the detection and quantification of the single toxins as well as the understanding of NheABC interaction required for the cytotoxic action. By the means of these tools it could be shown that: i) NheC forms complexes with NheB in the supernatants of *B*. *cereus* cultures [20]; ii) a specific binding order is required [21], with NheC or NheB/C being mandatory for target cell priming; iii) a molar ratio of approx. 10:10:1 (NheA:NheB:NheC) is important for maximum toxicity [22].

These studies further underlined, that NheA is mandatory in the final step of pore formation. Earlier attempts to generate monoclonal antibodies towards the A component of the Nhe-toxin complex have resulted in the availability of mAb 1A8 [17] and 2G11 (unpublished data). In contrast to the present approach, purified NheA from *B*. *cereus* supernatants was used as immunogen. This earlier immunization resulted in the generation of 7 hybridoma cell lines. Both antibodies named above exhibit several disadvantages concerning their applicability for detailed studies on NheA. Major drawbacks namely are i) lack of neutralization capacities (1A8) ii) lack of reactivity towards NheA in solution (1A8) iii) weak reactivity towards rNheA (1A8) iv) weak reactivity against NheA from *B*. *cereus* supernatants (2G11) in an indirect EIA. The present study aimed to further proceed with the characterization of the A component by novel monoclonal antibodies exhibiting the required properties.

Due to the neutralization capacities of one of the newly established antibodies a direct visualization of NheA in the full complex on the target cells was enabled. Furthermore, a highly sensitive sandwich EIA for NheA quantification in *B*. *cereus* supernatants was established. Together with the results from epitope-mapping experiments it could be concluded that: i) NheA is a flexible molecule; ii) no interaction between NheA and NheB or -C occurs in solution iii) cell-bound NheB is the main binding partner for the A-component and iv) a conformational rearrangement of the A-component finalizes the pore-forming process.

## Material and Methods

### Ethics Statement

Animal husbandry and experiments for the generation of monoclonal antibodies were conducted according to the German Law for Protection of Animals. Study permission was received by the Government of Upper Bavaria (permit number: 55.2-1-54-2531.6-1-11).

### Bacterial Strains

Cell-free supernatants of MHI 241 (Nhe reference strain NVH 0075/95), DSM 31, MHI 1761 (producing NheB and NheC only) and other strains of the in-house collection were applied in the present study. The main characteristics of these strains are summarized in S1 Table. Toxin expression was performed in casein hydrolysate glucose yeast (CGY) broth supplemented with 1% glucose as described elsewhere [23].

### Expression of Recombinant NheA (rNheA)

For immunogen preparation, *nheA* from DSM 31 without the original stop codon was amplified by PCR (NheAfor: 5’ caccgcgcaaaatgtaattgc; NheArev: 5’ atgtgcttcaacgtttgtaacgtaatc) and cloned in pBAD202 vector (Invitrogen, USA). After confirmation of the sequence integrity (GATC Biotech, Gemany), an arabinose responsive *E*. *coli* strain (LMG194) was transformed and recombinant NheA was expressed according to the manufacturer’s protocol. The resulting recombinant protein is characterized by an N-terminally located thioredoxin tag that can be removed by enterokinase (Sigma, Gemany) digestion and a C-terminally hexa-histidine tag for purification on Ni^2+^-agarose. Batch purification was carried out according to the manufacturer’s instructions (Qiagen, Germany). The protein concentration in the eluate fraction was determined on SYPRO ruby (Invitrogen, USA) stained SDS-PAGE (G&E Healthcare Mini-System, USA) using BSA (Sigma, Germany) as standard. For cell-based assays (cytotoxicity, flow cytometry and immunofluorescence microscopy) NheA containing the original stop codon was cloned into pASK IBA5+ (IBA GmbH, Gemany) and expressed via an anhydrotetracycline inducible promoter in DH5-alpha cells. This approach resulted in an N-terminally Strep-tagged protein that was purified by StrepTactin sepharose columns (IBA GmbH, Germany) according to the manufacturer’s recommendations.

### Generation of N-Terminally Truncated rNheA

N-terminally truncated *nheA* fragments were amplified by PCR using the primers NheA-109for 5’: ttacaggtacctcaagaaatgatgagatacagc; NheA-164for 5’: tttacggaggtacctttagagttaaatcgatttaaaacag; NheA-185for 5’: taccataggtacctgatgaagcaataaaaacactgc and NheArev 5’: ttggcgagagtcgacttaatgtacttcaacg containing a KpnI and SalI restriction site, respectively. After restriction digest the fragments were ligated with the pASK IBA5+ vector. Next to transformation of DH5α and sequence conformation (GATC Biotech, Gemany) expression of N-terminally deleted NheA and Strep-Tag purification was performed.

### Generation of Monoclonal Antibodies (mAbs)

Purified rNheA in PBS, adjusted to aliquots of 30 μg rNheA per animal and emulsified in Sigma adjuvant, was injected in five 12 weeks old female hybrid mice [BALB/c x (NZW x NZW)]. Booster injections were given at day 40 and 140. At day 180 (three days), before cell fusion mice received a final booster with 45 μg rNheA in PBS per animal. Immunization of mice, cell fusion experiments, establishment of hybridomas and antibody purification were performed according to Dietrich *et al*. 1999 [24]. Hybridoma cultures were assayed for secretion of NheA reactive antibodies by indirect EIA using rNheA or *B*. *cereus* culture supernatants as coating antigen. A total of 15 anti-NheA secreting hybridomas were received.

### Characterisation of mAbs

Received mAbs were assayed for their specificity, sensitivity as well as Ig subtype. The latter was carried out by mouse hybridoma subtyping kit (Sigma, Germany) according to the manufacturer’s recommendations. The mAb 1G4 as well as 1A8 and 2G11 were further subjected to peptide-based epitope mapping (PEPperPrint BioCat GmbH, Germany). In principle, this technique is based on a microarray chip spotted with 370 overlapping 13-meric peptides covering the protein used as immunogen. For detailed methodological information contact the PEPperPrint Company. The capability to react with NheA in solution was assayed in a competitive EIA format and in case of mAb1G4 by immunoaffinity chromatography (IAC). In the competitive EIA serially diluted NheA was coated on the plates overnight. On the next day 2 μg/ml of the mAb were competitively co-incubated with a 2-fold molar excess of NheA for 30 min and then added to the plates. For the negative control the antibodies were exposed to PBS or a crude lysate obtained from the *E*. *coli* expression line (DH5α). All other blocking and detection steps were similar to the procedure applied for the indirect and sandwich EIAs.

### Indirect and Sandwich EIAs

For indirect EIAs, microtiter plates (Maxisorb Nunc, Germany) were coated with serial dilutions of *B*. *cereus* supernatants or rNheA overnight at room temperature. Plates were blocked with 3% [w/v] casein-PBS for 30 min. Primary antibodies were added (1 μg ml^-1^) for1 h at room temperature; then a horseradish-peroxidase (HRP)-labeled anti-mouse conjugate was applied at 1:2,000 for 1h at room temperature. Incubation with 3,3′,5,5′-tetramethylbenzidin (TMB) substrate was performed for 20 min and reaction was stopped by 1 M H_2_SO_4_. OD recording was done on a TECAN (Switzerland) EIA reader (450 nm with 620 nm reference filter).

In order to establish a sandwich EIA for the quantitative detection of NheA each of the mAbs was tested for capture or as an HRP-conjugate for detection. With exception of mAb 1G4-HRP (lost reactivity after labeling) antibodies were tested by coating 2.5 μg ml^-1^ of the capture antibody in PBS over night at room temperature to the plates. After blocking with 3% [w/v] casein-PBS serial dilutions of rNheA or supernatants in 0.5% [v/v] Tween-PBS were added for 1h at room temperature. Next, the HRP-labeled detection antibody was applied (1:2,000 in 1% [w/v] casein-PBS for 1 h). Substrate addition and signal recording was performed as described above. A NheB-specific sandwich EIA was performed in order to quantify the NheB concentration in the *B*. *cereus* supernatants used for cytotoxicity, neutralization, immunofluorescence microscopy and flow-cytometry experiments as described earlier. This EIA includes the mAb 2B11 for capture and 1E11-HRP for detection [25].

### Immunoaffinity Chromatography (IAC)

For preparation of the IAC column, 10 mg mAb 1G4 were coupled to 1 g of CNBr-activated Sepharose 4b according to the manufacturer’s instructions (GE Healthcare, USA). The initial sample (supernatant) of MHI 241 and all fractions collected during the immunoaffinity purification procedure were assayed in an indirect EIA using the earlier described mAb 1A8. Retrieved reciprocal EIA titers were termed NheA units. By multiplying NheA units with the volume of the corresponding fraction, volume-corrected total units could be calculated.

### Western-Blot

Immunoreactivity of the novel mAbs under denaturing conditions was tested by Western-blot analysis. Full length rNheA or the N-terminally deleted NheA fragments as well as supernatants of *B*. *cereus* strains were run on 10–15% Minigel (G&E Healthcare, USA) followed by blotting on a PVDF-P membrane (Merck Millipore, Germany) and blocking with 3% casein-PBS. Primary antibodies (2 μg ml^-1^) were incubated for 1 h at room temperature. After washing, an HRP-labeled anti-mouse antibody (1:2,000, Dako, Germany) was added for 1 h. Chemiluminescence signals induced by addition of Super Signal Western Femto substrate (Pierce, USA) were recorded on a Kodak image station (Eastman Kodak, USA).

### Neutralization of *In Vitro* Cytotoxicity

All assays were carried out on Vero (ATCC^®^ CCL-81^™^, USA) cells maintained in MEM medium (Biochrom AG, Germany) supplemented with 1% FCS (Sigma), 1% Na-Pyruvat (Biochrom AG, Germany) according the suppliers recommendations. Pen/Strep (Biochrom AG) was added to avoid potential contamination by non-sterile recombinant proteins or *B*. *cereus* supernatants. Cell number was adjusted to 15,000 per well. Metabolic activity of the cells was recorded after supplementation of 10 μl/well WST-1 (water soluble tetrazolium salt, Roche, Germany). This assay is based on the NADH- dependent bioreduction of the WST-1 reagent to formazan and thus an indicator of mitochondrial activity. A TECAN EIA reader served for OD measurement at 450 nm with 620 nm reference filter. The 50% cytotoxic dose was then calculated.

In case of *B*. *cereus* supernatants serial dilutions were added to the cells after 30 min pre-incubation with a constant amount (10 μg/well) of the antibodies under study. An anti-3AcDON mAb was applied as isotype control. For the consecutive neutralization approach, NheB/C was added at a fixed concentration of 100 ng ml^-1^ NheB as determined by a NheB-specific sandwich-EIA and allowed to rest on the cells for 2 h. After a washing step neutralized 100 ng ml^-1^ rNheA was serially diluted and added to the primed cells. Cytotoxicity titers were determined as described above.

### Flow Cytometry

Flow-cytometry assays were performed to show the interaction of NheA with NheB/C primed cells only. All experiments were carried out in triplicates on a FACS Calibur (BD BioScience, USA) using the CellQuestPro software (BD Bioscience, USA) for data recording. For the assays, trypsinated Vero cells were adjusted to 1,000,000 cells ml^-1^ in EC buffer (140 mM NaCl, 15 mM HEPES, 1 mM MgCl_2_, 1 mM CaCl_2_, 10 mM glucose pH 7.2). Supernatants of MHI 241 were incubated with 10 μg ml^-1^ mAb 1G4 or 2G11 30 min before addition to the cells. In further experiments, serial dilutions of NheB/C (MHI 1761) complemented with a constant concentration of antibody neutralized rNheA were incubated on the cells for 1 h at 37°C with constant agitation. Flow-cytometry is only feasible on suspension cells. The adherent Vero cells had to be trypsinated prior to the treatment, because the Nhe components are protease sensitive and would not have been detectable after trypsinization.

To further assay the interaction of NheA with NheBC on the cellular level, the molar ratio of NheB:C in MHI 1761 supernatant was adjusted to 1:1 by addition of extra rNheC followed by incubation with neutralized rNheA. After these different experimental set-ups, cells were washed two times in 5% [w/v] BSA-PBS and then incubated with goat-anti-mouse Alexa-488 antibody (2 μg/ml) for 1 h at 4°C. Finally, cells were washed three times in PBS, diluted in FACS buffer (1% [w/v] BSA-PBS) and subjected to flow cytometry.

### Immunofluorescence Microscopy

For direct visualization of cell-bound NheA, immunofluorescence microscopy was carried out in 8-well Lab-Tek chamber slides (Nunc) seeded with 60,000 Vero cells per well. Supernatant of MHI 1507 (adjusted to 100 ng ml^-1^ NheB) was pre-incubated with mAb 1G4 (10 μg/well) 30 min prior to addition to the cells in order to neutralize cytotoxic effects. MHI 1761 (adjusted to 100 ng ml^-1^ NheB) was applied on Vero cells together with a constant amount of NheA (200 ng ml^-1^) neutralized by 1G4 (10 μg/well). Neutralized components were incubated for 60 min. After 3 times washing in PBS, cells were fixed in ice-cold methanol for 10 min at -20°C. Methanol was removed by 3x washing in PBS and cells were blocked in 1% BSA-PBS containing 5% goat-serum for 30 min. Afterwards another 10 μg/well of mAb 1G4 had to be added to the cells as the antibody from the neutralization step was removed during fixation and was therefore no longer detectable by goat-anti-mouse Alexa-488 labeled conjugate (5 μg ml^-1^ for 1 h; Molecular Probes, USA). After thoroughly washing nuclei were counterstained with DAPI (Invitrogen, USA). Images were recorded on a Zeiss LSM 510 microscope (Carl Zeiss GmbH, Germany).

### Statistics

Calculations of means, standard deviation or variation coefficient have been performed in Microsoft Excel 2010 (Microsoft Corp., USA) or in case of graph preparation in GraphPad Prism 5.04 (GraphPad Software Inc., USA)

## Results

After immunization of mice with His-tag purified, recombinant NheA containing an N-terminal thioredoxin tag 15 hybridoma cell lines were established and thoroughly tested with regards to their characteristics. It has to be taken into account that the immunogen includes an artificial thioredoxin tag which could probably provoke an undesired antibody response. Furthermore, high sensitivity binding and broad spectrum applicability of the antibodies in diverse assay systems had to be ensured. The mAbs 1A4 and 1F6 were excluded from the further experiments due to their disadvantages summarized in S1 Fig.

### Comparison of mAb 1A8, 2G11 and 1G4 Immunoreactivity

Table 1 depicts the characteristics of the antibodies applied. The mAb 1G4 showed a detection limit of 107 ng ml^-1^l rNheA in an indirect EIA (resulting in an OD_450_ of 0.3). A similar EIA sensitivity was found for the mAb 2G11 (98 ng ml^-1^). In contrast, mAb 1A8 which was initially obtained after immunization with natural NheA does not react with rNheA. None of the antibodies under study exhibits cross-reactivity towards NheB or NheC. Natural NheA from *B*. *cereus* culture supernatants was detectable by mAb 1A8 as well as by mAb 1G4. In contrast to the mAb 1A8, titers retrieved by 1G4 differed depending on the strain under study. This is reflected by a variation coefficient of 48% for mAb 1G4 and 16% for mAb 1A8 respectively.

**Table 1:** Overview on the most important characteristics of the three mAbs against NheA applied in the present study.

mAb	Ig subtype	sensitivity rNheA^#^	Inhibitions of antibody reactivity by soluble antigen	Titer* in indirect EIA (strain no.)						
				MHI163	MHI165		MHI241	DSM31	NVH 1280/33	
1G4	IgG_2a_	107 ng ml^-1^	63%	1:103 (± 12)	1:30 (± 10)	1:39 (± 5)		1:98 (± 13)		1:94 (± 17)
1A8	IgG_1_	negative	negativ	1:760 (± 13)	1:870 (± 1)	1:861 (± 3)		1:631 (± 15)		1:974 (± 3)
2G11	IgG_2a_	98 ng ml^-1^	42%	1:10 (± 3)	1:7 (± 5)	1:15 (± 10)		n. a		n. a

^#^ sensitivity defined as the concentration of rNheA resulting in an OD_450_ of 0.3 under the conditions of an indirect EIA; values in brackets indicate the variation coefficient in %.

n. a. not analyzed

* dilution resulting in an OD_450_ of 1.0

These results gave a first hint that strain-specific differences e. g. expression rates or sequence alterations in the epitope region might contribute to the alternate results. The latter assumption was tested by an epitope mapping approach (results presented below). In an indirect EIA the reactivity of mAb 1G4 could be reduced by 63% when the antibody was competitively co-incubated with NheA. A 42% reduction in EIA-reactivity was achieved for mAb 2G11 during a competitive co-incubation. Interestingly, for mAb 1A8 the competition failed.

The binding capability of mAb 1G4 towards NheA in solution was corroborated in further studies. Herein, the protein was purified from *B*. *cereus* supernatants by immunoaffinity chromatography (IAC). More than 90% of the loaded NheA was bound by the IAC column and approx. 74% of the column bound NheA could be retrieved in the eluate fractions (Table 2). The eluate fraction was also tested for the presence of proteins putatively interacting with or binding to NheA. However, neither NheB nor NheC were detectable.

**Table 2:** Results of the mAb 1G4-based IAC purification of NheA from *B*. *cereus* MHI 241 supernatants.

Fraction	NheA units^1^ (reciprocal EIA titer)	Volume (ml)	total units	% NheA
sample	1,436	15 ml	21,542	100
flow through	80	15 ml	1212	5.62
wash	20	30 ml	600	2.8
eluate 1	1,576	10 ml	15,760	73.2
eluate 2	14	10 ml	143	0.66

^1^ determined by indirect EIA using mAb1A8

**Fig 1:**
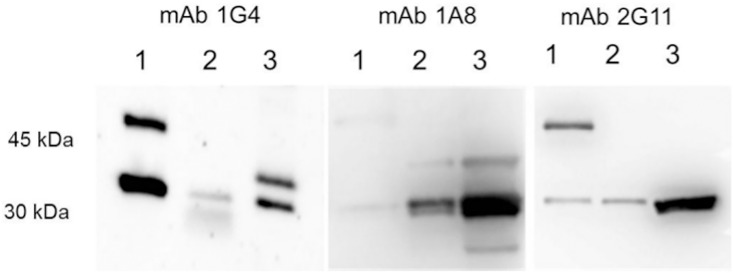
Reactivity of NheA-specific antibodies 1G4, 1A8 and 2G11 in a Western-blot assay. Protein lysates applied were as follows: rNheA (1) supernatant of MHI 241 (2) supernatant of MHI 241 concentrated (3). The concentration was performed on Amicon^®^ (Merck Millipore, Germany) Ultra centrifugal filters with a size exclusion of 30 kDa. The upper band in lane 1 represents rNheA with the 13 kDa thioredoxin tag still attached.

The antibodies were further tested for their performance in Western-immunoblotting. 1G4 reacts well with enterokinase digested rNheA and to a lesser extent with natural NheA. In contrast to the indirect EIA, mAb 2G11 detects natural NheA under the denaturing conditions of a Western-Blot. Detection of recombinant NheA by mAb 1A8 failed (Fig 1).

### MAb 1G4 Enabled NheA Detection in a Sandwich EIA

**Fig 2:**
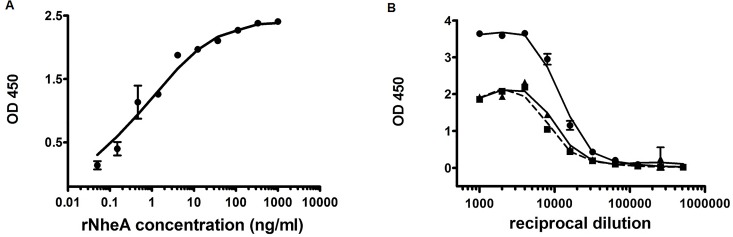
Establishing a NheA specific sandwich EIA. (A) Standard curve for the quantification of rNheA by the highly sensitive sandwich EIA in which mAb 1G4 served as capture and 2G11-HRP as detection antibody. The detection limit is as low as 20 pg/ml. (B) Exemplified reactivity of toxin containing supernatants from *B*. *cereus* strains in the NheA-specific sandwich EIA; MHI 1507 (dot); MHI 241 (square) and MHI 1308 (triangle up). The samples were assayed in duplicates and depicted as means and standard deviation (SD).

All three NheA reactive antibodies and especially the mAb 1G4 were next tested in order to establish a sandwich EIA. Different combinations of antibodies either as capture or as HRP-labeled detection tool were assayed. Any combination comprising mAb 1A8 failed. Unfortunately, the combination of mAb 2G11 for capture and mAb 1G4-HRP for antigen detection could not be assayed as the latter antibody lost its reactivity during the HRP-labeling reaction. Combining mAb 1G4 for antigen capture and HRP-labeled mAb 2G11 for antigen detection resulted in a highly sensitive sandwich EIA with a detection limit of approx. 20 pg ml^-1^. The standard curve is depicted in Fig 2*A*. Fig 2*B* shows the performance of *B*. *cereus* supernatants in the novel sandwich EIA.

In parallel samples were assayed in a NheB-specific sandwich EIA and by cytotoxicity assays (Table 3). This EIA comprises the mAb 2B11 for capture and mAb 1E11-HRP for antigen detection as described earlier [25]. A quantification of the NheC contents is not possible. More than 90% of the C-component is complexed with NheB and to date no EIA is available for NheC quantification. However, the NheB/C-complexes are detectable in a semiquantitative matter as published elsewhere [20].

**Table 3:** Concentration of NheA and NheB in cell-free supernatants of different *B*. *cereus* isolates. Seven out of 12 tested supernatants show similar concentrations for NheA and B as it has been proposed by Lindbäck et al. [22]. Two strains expressed two-fold higher NheB amounts (highlighted with light red background), whereas in two strains a three-fold higher NheA (highlighted with light green background) concentration was detected.

strain no.	NheA (μg/ml)	NheB (μg/ml)	Cytotoxicity titer (50% Tox.)
MHI 241	4.8 (± 1.48)	10.6 (± 1.27)	1:674
MHI 1477	4.9 (± 0.92)	10.4 (± 0.28)	1:1220
MHI 1503	5.9 (± 0.07)	3.8 (± 0.14)	1:892
MHI 1507	8.2 (± 3.32)	8.7 (± 0.07)	1:428
MHI 1522	1.5 (± 0.02)	1.3 (± 0.03)	1:316
MHI 1561	3.0 (± 0.49)	2.2 (± 0.07)	1:474
MHI 1661	3.5 (± 1.48)	3.4 (± 0.71)	1:572
MHI 1692	5.5(± 0.49)	7.0 (± 0.42)	1:624
MHI 2963	14.2 (± 0.21)	12.6 (± 0.21)	1:1064
MHI 2964	8.9 (± 0.28)	2.8 (± 0.14)	1:1482
MHI 2967	5.3 (± 0.49)	3.8 (± 0.71)	1:1124
MHI 2972	8.1 (± 1.56)	2.0 (± 0.28)	1:1563

values in brackets show the standard variation

Cytotoxicity of strains showing a diverging NheA:B ratio does not significantly differ from isolates with a ratio of approx. 1:1. Comparison was performed by T-test (p> 0.05).

### Epitope Mapping

**Fig 3:**
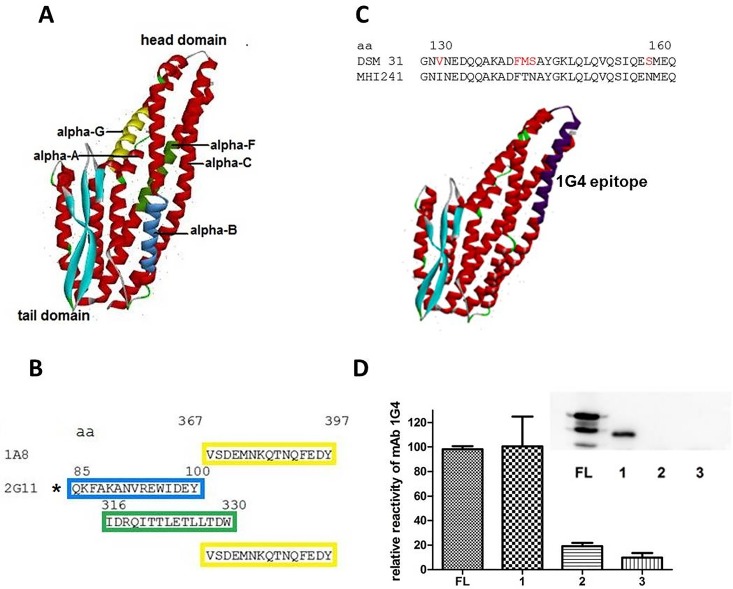
Epitope mapping results. (A) NheA structure according to pdb file 4K1P with colored positions of the epitope mapping results. (B) amino acid (aa) numbers and sequences of epitopes for 1A8 (single epitope) and 2G11 (three potential binding sites the C-terminal one being identical to that of 1A8). The frame colors correspond to the colored structures in (A).The asterisk marks the likeliest epitope according to the results presented in Table 4. (C) Aa exchanges in MHI 241 compared to the type strain DSM 31 between positions 130 and 162 and location of the putative 1G4 binding site (dark purple), (D) 1G4 reactivity in indirect EIA with N-terminally deleted rNheA (FL—full length NheA, bar 1 – rNheA ^-109^ aa, bar 2 – rNheA ^-164^ aa, bar 3 – rNheA -^185^ aa. Means and SD in the depiction represent triplicate measurements. The insert shows the corresponding Western-blot results performed with mAb 1G4 and rabbit anti-mouse-HRP conjugate for detection of the recombinantly expressed NheA and its fragments.

Fig 3*A* and 3*B* depict the results obtained from the peptide-based epitope mapping. For mAb 1A8 one epitope at the very C-terminus of NheA could be determined. Three potential binding sites for mAb 2G11 were suggested, the very C-terminal one being identical to that of 1A8. Unfortunately, peptide mapping for the mAb 1G4 failed. Therefore three recombinantly expressed N-terminal deletion mutants (rNheA^-109,-164 and -185^) were generated and tested. This decision was based on the observation that this antibody exhibits a more than 50% reduced reactivity towards MHI 241 compared to rNheA from DSM 31 (see: Table 1) which was used as immunogen. Sequence comparison of NheA from both strains revealed several amino acid exchanges at positions 130-162. These might contribute to the different performance (Fig 3*C*). Results of the indirect EIA and Western-blot are depicted in Fig 3*D* and show that deletion of 109 aa from the N-terminus does not reduce the antibody reactivity. Hence, deletion of 164 and 185 aa resulted in a reduction of approx. 80 and 90% EIA reactivity respectively. For rNheA^-164^ and rNheA^-185^ no reactivity in Western-blot was found.

**Table 4:** Reactivity of the recombinant NheA fragments in different EIA set-ups substantiate the epitope-mapping experiments based on synthetic peptides and allow the allocation of the 1G4 and 2G11 epitope.

antibody	EIA set-up	antigen			
		NheA FL	NheA-109	NheA-164	NheA-185
1G4	indirect	**+**	**+**	*	*
2G11	indirect	**+**	**-**	**-**	**-**
1G4/2G11-HRP*	sandwich	**+**	**-**	**-**	**-**

* remaining EIA reactivity approx. 10–20%

These results allocate the binding site between aa 109 and 164 according to the reactivity towards the N-terminally deleted proteins—preferably between 130 and 164 according to the sequence comparison.

Based on the truncated rNheA the epitope specificity of mAb 2G11was reanalyzed. In an indirect EIA format this antibody reacts only with the full length sequence. When the sequentially deleted rNheA fragments were assayed in the established sandwich EIA only the full length protein could be detected (data not shown). Even at concentrations up to 24 μg ml^-1^ the sandwich EIA showed negative results thus pointing to the suggested N-terminal epitope (aa 85–100). Table 4 summarizes the different reactivity of mAb 1G4 and 2G11 the three EIA set-ups.

### MAb 1G4 and 2G11 but Not 1A8 Neutralize Nhe-Related Cytotoxicity

**Fig 4:**
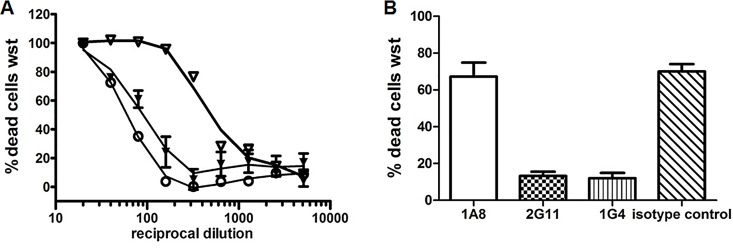
Results of the neutralization assays. (A) Neutralization of toxic activity in supernatants from MHI 1507 by mAb 1A8 (open triangle), mAb 2G11 (black triangle) and mAb 1G4 (open dot). A high reciprocal dilution at the 50% toxic dose indicates a low or absent neutralizing capacity of the antibody applied. The neutralizing effect of 1G4 and 2G11 are similar while 1A8 and the isotype control (see S2 Fig) are not able to neutralize the cytotoxicity. (B) Consecutive neutralization of serially diluted antibody treated rNheA (applied to NheB/C (MHI 1761) primed cells. With mAb 1A8 or the isotype control the majority of the cells is dead. Error bars represent the SD of triplicates.

The overall neutralization capacity was assayed by testing serial dilutions of Nhe-containing supernatants of MHI 241 and MHI 1507 and addition of a constant amount (10 μg/well) of either mAb 1G4, 2G11, 1A8 or an antibody reactive towards 3AcDON for isotype control. Results of the neutralization assays are given in Fig 4*A*. S2 Fig depicts the cytotoxicity curves for mAb 1A8 and the isotype control clearly showing the lack of 1A8 neutralization. The mAb 1G4 is able to neutralize more than 90% of the Nhe-related cytotoxicity. Corresponding titers are: 1:53 vs. 1:725 for MHI 241 and 1:40 vs. 1:465 for MHI 1507, respectively. Unexpectedly, mAb 2G11 exhibits a neutralization capacity towards NheA similar to that of 1G4 although the antibody 2G11 weakly binds to NheA from *B*. *cereus* supernatants in an indirect EIA. A further consecutive neutralization approach including the solely NheB/C expressing strain (MHI 1761) in the priming step and neutralized rNheA in the second step (Fig 4*B*) was carried out. Under these reaction conditions, mAb 1A8 again failed to neutralize the cytotoxic effects. Prevention of pore-formation due to the application of mAbs 1G4 and 2G11 has additionally been demonstrated by propidiumiodide influx assays (S3 Fig).

### Flow Cytometry Uncovers Different Neutralization Mechanisms for mAb 1G4 and 2G11

**Fig 5:**
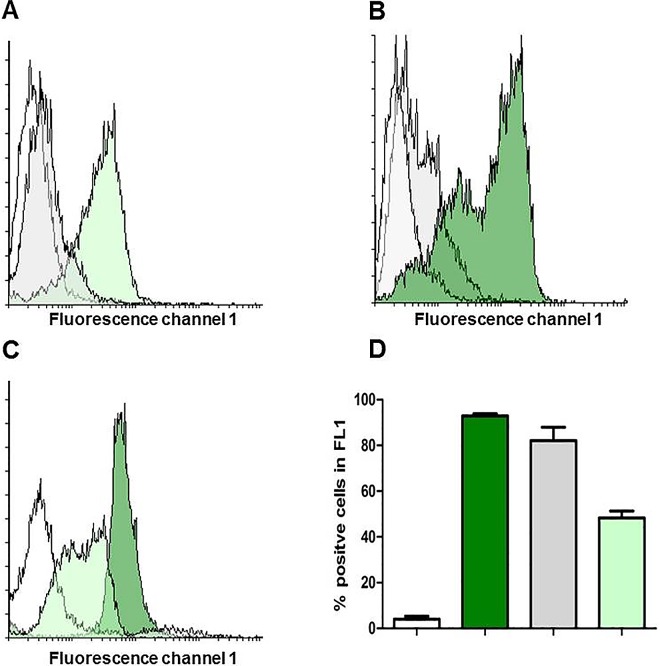
Neutralization effects assayed by flow-cytometry. (A) and (B) show the different neutralization capacities of mAb 1G4 and 2G11. (A) supernatant of MHI 241 was neutralized by mAb 1G4 (green histogram) or mAb 2G11 (grey histogramm. (B) NheBC was incubated with 1G4 (green histogram) or 2G11 (grey histogram) neutralized rNheA. White histograms represent the negative controls (Vero cells incubated with primary and secondary antibodies. (C) overlay histogram after incubation of Vero cells with NheBC (adjusted to NheB at 150, 75 and 50 ng ml^-1^ and approx.15, 7.5 and 5 ng/ml^-1^ NheC, respectively) together with a constant amount of rNheA (150 ng ml^-1^) neutralized by mab 1G4; white = negative control; light green = NheB—50 ng/ml; dark green = NheB 150 ng/ml for reasons of overview results obtained with 75 ng/ml^-1^ NheB have only be included in the bar chart in (D); (D) means and SDs for NheA cells positive in fluorescence channel 1; colors correspond to those in the overlay histogram (C).

Because the mAbs 1G4 and 2G11are both able to neutralize Nhe-related cytotoxicity and are reactive towards different epitopes, we next investigated the neutralization mechanism on the cellular level. In flow cytometry NheA from the supernatant of MHI 241 could only be detected on 13% (± 0.8) of the Vero cells when mAb 2G11 was applied for neutralization. This finding contrasts the results obtained with mAb 1G4. With the latter antibody NheA is still detectable on approx. 85% (± 4.2) of the cells (Fig 5*A*). Similar results were obtained when rNheA was first neutralized and then combined with the NheB/C supernatant (Fig 5*B*). In this consecutive approach the inhibition of rNheA binding to NheBC by mAb 2G11 could be confirmed. Approx. 13% (± 2.6) of the cells were positive for NheA with mAb 2G11applied. In contrast, NheA was present on 85% (± 0.7) of the cells when mAb 1G4 was added. It could therefore be concluded that the mechanisms of neutralization seem to be different for mAb 1G4 and 2G11. The mAb 1G4 clearly blocks the final pore formation whereas mAb 2G11 hinders the attachment of the A-component to NheB/C.

### NheA Is Detectable on the Cell Surface Only in the Presence of NheB/C and in a NheB/C Concentration-Dependent Matter

The mandatory presence of NheB/C prior to NheA binding was demonstrated in additional flow cytometry experiments. When cells were pre-incubated with varying NheB/C concentrations and neutralized rNheA was added at a constant concentration, the amount of NheA positive cells clearly depends on the NheB/C amount added during the first step (Fig 5*C* and 5*D*). No NheA binding was observed when the cells were pre-incubated with rNheC only or after single addition of rNheA (S4 Fig).

**Fig 6:**
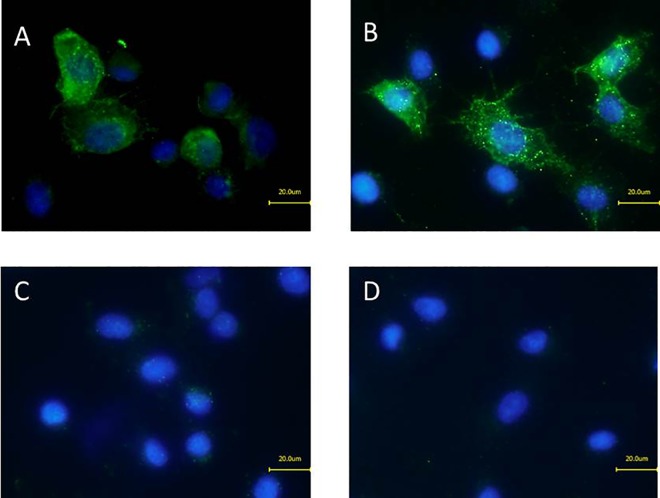
Immunofluorescence microscopy of NheA on Vero cells. Staining of cell-bound NheA after treatment of Vero cells with *B*. *cereus* supernatants. (A) MHI 1507 NheB (100 ng ml^-1^ and approx. 10 ng ml^-1^ NheC) with mAb 1G4 neutralized NheA untreated Vero cells stained with primary and secondary antibody. (B) MHI 1761 (containing NheB at 100 ng ml^-1^ and approx. 10 ng ml^-1^ NheC) supplemented with neutralized rNheA (200 ng ml^-1^). (C) Stained Vero cells treated with rNheA only. (D) Untreated Vero cells stained with primary and secondary antibody. All slides were counterstained with DAPI.

Presence of neutralized, cell-bound NheA in the full toxin-complex was further assayed by immunofluorescence microscopy. Results were in accordance with those received in flow cytometry. Single staining of NheA from neutralized supernatant of MHI 1507 in an indirect approach using mAb 1G4 and goat anti-mouse-Alexa-488 resulted in a green fluorescence signal as shown in Fig 6*A*. In further experiments cells were pre-incubated with NheB/C from MHI 1761. Consecutively neutralized rNheA was added and staining was performed. This also resulted in a NheA-specific fluorescence (Fig 6*B*). In contrast, cells incubated with rNheA only did not show a fluorescence signal (Fig 6*C*). Vero cells treated with the primary and secondary antibody reacted correctly negative (Fig 6*D*).

### A NheB:C Ratio of 10:1 Is Necessary for NheA Binding

**Fig 7:**
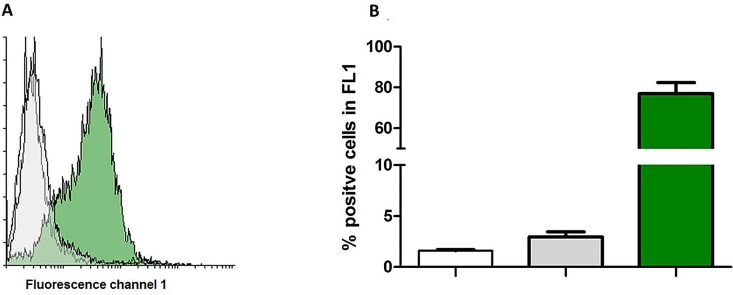
NheA binding to NheB/C adjusted for different ratios and assayed by flow-cytometry. (A) Expemplified overlay histogramm of Vero cells incubated with a NheB/C ratio of 10:1 (green histogram) and 1:1 (grey histogramm) followed by addition of 1G4 neutralized rNheA. The artificial adjustment of NheB/C ratio to equal levels will result in the formation of NheB/C heterodimers that recruit less NheA compared to the 10:1 ratio. Cells incubated with neutralized rNheA only are depicted in the white histogramm. (B) bar charts generated according to the flow cytometry experiments with different NheB/C ratios; the colours of the bars correspond to the set-ups in (A). Error bars represent the SD of triplicates.

Previous studies have shown that a defined ratio of NheB:C (10:1) is a prerequisite for maximum NheB binding, pro-pore formation and thus high cytotoxicity [20,26]. The importance of the above mentioned ratio for NheA binding could not be investigated so far due to the lack of suitable antibodies. With the availability of mAb 1G4 this problem was solved. It could be shown that an increasing amount of extra rNheC resulting in a ratio of NheB:C of 1:1 not only leads to a reduced presence of NheB as described earlier [20] but also influences the NheA presence on the cells. At the natural NheB/C ratio approx. 80% (±5.7) of the cells are NheA positive whereas at the artificially adjusted ratio of less than 5% of the cells are positive. For the depiction of the results, see: Fig 7*A* and 7*B*.

## Discussion

Several antibody-based systems have been established and facilitated the detection, quantification and neutralization of *B*. *cereus*-associated Nhe toxins especially NheB and -C [20,21,27]. Thereby, important knowledge on the mode-of-action of this three-component toxin-complex could be gained. However, the role of NheA in the ultimate step of pore-formation remains unclear. By a thorough characterization of three monoclonal antibodies we were able to establish a highly sensitive sandwich EIA in order to quantify NheA in culture supernatants. Several antibody-binding studies combined with peptide-based epitope mapping enabled us to understand the conformational flexibility of the NheA component. Due to the neutralization capability of mAb 1G4, NheA could—for the first time—be directly detected on target cells in the tripartite full-complex.

### Distinct Reactivity Pattern of the mAbs Indicate the Flexibility of NheA

Taking into account the diverging reactivity of the antibodies in various immunochemical assay systems it can be concluded that the conformation of NheA is flexible. Depending on whether it is bound to a surface or is present in solution the exposure of antigenic sites seems to differ. The phenomenon of altered exposure for target-bound versus unbound structures has recently been demonstrated for the HI-virus envelope glycoprotein gp120 [28,29]. Furthermore, alteration of protein structure and function has also been shown upon binding to nanoparticles [30]. The fact that mAb 1A8 only binds to NheA coupled to a solid surface, e. g. a microtiter plate suggests that the 1A8 epitope is not accessible until NheA binding occurred. Thus a conformational transition of bound vs. unbound NheA can be assumed. According to the epitope-mapping results for mAb 1A8 this transition occurs at the C-terminus of NheA. Due to the lack of neutralization capacities the assumed molecular alteration of NheA after binding to NheB/C on the target cells could not be assayed by the means of mAb 1A8. In contrast, the antibody binding region of mAb 1G4 is obviously accessible on surface bound and unbound NheA molecules as substantiated by various immunochemical methods applied. The diverging EIA reactivity of mAb 2G11 towards NheA in supernatants vs. rNheA (Table 1) is, additionally, an interesting feature. Initially the latter antibody had only been tested with NheA from *B*. *cereus* supernatants and showed only very weak reactivity in an indirect EIA. The different reactivity in the indirect EIA might indicate conformational differences in natural vs. recombinantly expressed NheA, eventually due to a slightly altered folding in *E*. *coli*. The results obtained from the different set-ups in order to establish a NheA-specific sandwich-EIA further underline the above suggested flexibility. The mAb 1A8 was neither successfully applied as a capture antibody nor as an HRP-labled detection tool when mAb 1G4 or 2G11 served for antigen capture. This observation is explainable by sterical hindrance of capture and detection antibody or binding of NheA to the capture antibody and thereby hindrance of the rearrangement needed for the 1A8 epitope exposure. As the epitope mapping experiments suggested a mutual binding site of mAb 1A8 and 2G11 at the C-terminus only 37 aa apart from a second potential 2G11 binding site (Fig 2*B*) a sterical hindrance seems possible. However, results of the N-terminally deleted NheA fragments in the indirect and the sandwich EIA clearly point a 2G11 epitope at the N-terminus of NheA. Thus an impaired conformational rearrangement of NheA after binding to mAb 2G11 or 1G4 seems more likely.

At a first glance, results obtained after testing of culture supernatants from 12 *B*. *cereus* isolates are partially contrasting earlier published results. On the protein level a molar ratio of 10:10:1 for NheA:B:C is necessary for maximum cytotoxicity [20]. This proposed ratio was not continuously detectable in the *B*. *cereus* strains under study (Table 3). A few strains showed a slightly higher (3:1) others a slightly lower (1:2) ratio of the NheA:B components. But one has to keep in mind that the binding efficiency of the capture antibody is influenced by the strain-specific amino acid sequence of the epitope, exemplified shown for DSM 31 vs. MHI 241 (Fig 3*C*). However, these diverging NheA:B ratios do not significantly influence the cytotoxicity titers (Table 3).

### Lessons to Learn from the Different Neutralization Properties

The mAb 1A8 does neither exhibit neutralization capacities towards NheA in natural supernatants nor towards rNheA. Assuming that the epitope is only accessible after a conformational rearrangement—in case of neutralization assays binding to NheBC—this final and crucial step of pore-formation cannot be reverted or arrested by mAb 1A8. Recently, it has been shown that the NheBC complex itself leads to the formation of permeable pro-pores [26] helping along full pore formation. Thus, terminal membrane damage due to attachment of NheA seems to outpace the potential binding of 1A8. Interestingly, mAb 2G11 is capable to neutralize both, natural as well as rNheA. Unexpectedly, mAb 2G11 exhibits neutralizing activity towards the naturally expressed NheA despite a weak performance in the indirect EIA (Fig 1 and Table 1). Comparing the results of the peptide epitope mapping in view of the diverging neutralization capacities it is likely that at least one of the suggested epitopes for mAb 2G11 is different from the common 1A8. As stated in the discussion of the sandwich EIA results it is highly likely that the sequence at the N-terminus (aa 85–100) comprises the 2G11 binding site.

The different reactivity pattern of mAb 1G4 vs. 2G11 whilst showing an equal neutralization effect prompted us to investigate the underlying mechanisms by the means of flow cytometry.

Results revealed different mechanisms of neutralization for mAb 1G4 and 2G11 (Fig 4*A* and 4*B*). MAb 2G11 it seems to hinder the penultimate step of pore-formation, i. e. attachment of NheA to the NheBC complex. Therefore the effect of 2G11 resembles that of the NheB reactive mAb 1E11 [27]. It can thus further be concluded that the N-terminus of NheA is important for attaching to cell-bound NheB/C.

In contrast, NheA is clearly detectable on the surface of Vero cells when 1G4 is applied for neutralization. Thus, the antibody neither sterically nor directly interferes with the binding of NheA to NheBC. Conclusively, the neutralization mechanism has to be caused by hindrance of the transition of the now formed NheABC complex to a full-pore. The inhibition of a NheA repositioning is further substantiated by the fact that a sandwich EIA could not be established by applying 1G4 for capture and 1A8-HRP as detection antibody. From the results discussed above it became clear that the latter one necessitates a conformational alteration-based epitope exposure prior to antigen binding. This is probably inhibited by attachment of NheA to mAb 1G4 applied for capture.

### What About NheA Partners in Solution?

The mAb 1G4 enabled IAC purification of NheA from *B*. *cereus* supernatants. This method is also suited to study co-elution of potentially interacting proteins in case of a strong and stable binding. Results clearly show that neither NheB nor NheC could be co-purified. This observation contrasts the earlier published results which suggest an interaction of NheA and -C when assayed on native SDS-PAGE [22]. However, in this study the proposed complexes could not be detected directly.

### Detection of NheA and Preferential Binding to NheB in the Full Complex

Based on the availability of the neutralizing mAb 1G4 NheA could further be detected on the target cells by flow cytometry (Fig 5*A* and 5*B*) and also by immunofluorescence microscopy (Fig 6*A* and 6*B*). Without the possibility of a neutralization step morphological damage such as cell rounding, detachment and disruption will occur as early as 15 min after incubation thus making the application of methods impossible which are in need of preserved cell morphology. NheA was not detectable on Vero cells in the absence of NheBC or in the presence of NheC only. This direct observation confirmed the indirect results obtained earlier by Western-blot [22]. Flow-cytometry additionally underlines that the concentration of NheBC applied determines the amount of NheA detectable of the target cells (Fig 5*C* and 5*D*). As the natural ratio of NheA:B:C for the achievement of maximum toxicity is assumed to be 10:10:1 [22] it seems reasonable that NheB is the main binding partner for NheA. Only at the natural ratio of NheB:C (10:1) more than 77% of the cells were positive for NheA (Fig 7*A* and 7*B*). This phenomenon is in accordance with the results published earlier. Herein, decreasing NheB amounts on Vero cells were shown when the ratio of NheB:C was increased, because less NheB is guided to the target cells [20].

**Fig 8:**
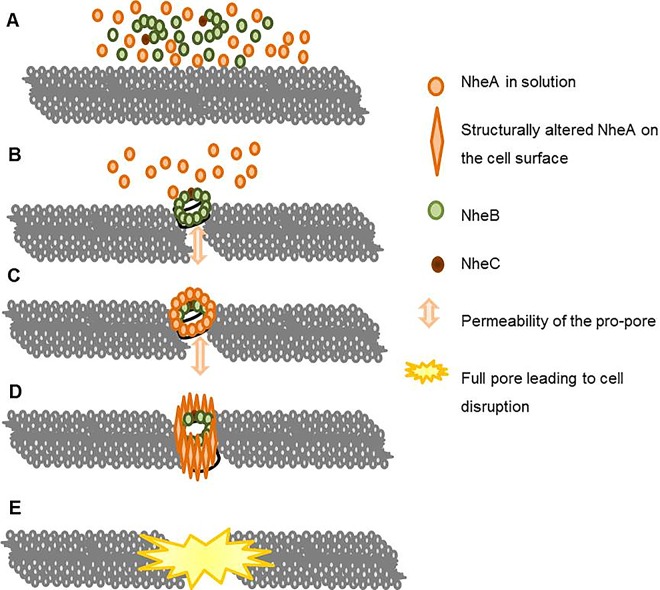
Schematic illustration of the refined pore-forming action of Nhe as substantiated in the present manuscript. (A) In solution NheA is present as single soluble molecules whereas NheB and -C form complexes as published [20]; (B) next the NheB/C complexes attach to the cell membrane where small permeable pro-pores are formed [26]; the pro-pore formation occurs independent of -A which is still in solution; (C) the penultimate step is the attachment of the A-component to the pro-pore followed by a conformational alteration (D) that leads to full pore-formation and destruction of the target-cell (E).

A high flexibility of NheA has always been suggested for the transition process from a small permeable pro-pore to a full pore. Summarizing the data presented herein and those from earlier publications [20,26,27], a five step procedure during the pore-formation can now be described (schematically summarized in Fig 8A–8E).

Firstly, in contrast to the complex forming NheB and -C, NheA is present as a single molecule in solution. Secondly, the NheB/C complexes attach to the cell membrane where small permeable pro-pores are formed. In the third step NheA attaches to the B-component. This step can be hindered by either NheB reactive mAb 1E11 or NheA reactive 2G11. Fourthly a conformational alteration of NheA occurs -preventable by mAb 1G4-, leading to an irrecoverable piercing of the target cell-membrane. This finalizes the pore forming process and seals the lethal fate of the cell in the fifth step.

## Supporting Information

S1 Fig Characteristics of the monoclonal antibodies 1A4 and 1F6 generated in this study. (DOCX)

S2 Fig Neutralization of toxic activity from MHI 1507 by mAb 1A8 and the isotype control. (DOCX)

S3 Fig Results of the PI influx assay. (DOCX)

S4 Fig Depiction of additional controls in flow cytometry. (DOCX)

S1 Table Nhe expression profiles of *B*. *cereus* strains relevant for this study. (DOCX)
