# Supplementary material for: Antibody Binding Studies Reveal Conformational Flexibility of the Bacillus cereus Non-Hemolytic Enterotoxin (Nhe) A-Component
Source: PLoS One. 2016 Oct 21;11(10):e0165135. doi: 10.1371/journal.pone.0165135 (PMC5074587; doi:10.1371/journal.pone.0165135)
Supplement: S1 Fig — (DOCX) [file pone.0165135.s001.docx]

**S1 Fig**

Characteristics of the monoclonal antibodies 1A4 and 1F6 generated in this study. The antibodies were also tested for cross-reactivity with NheB and NheC that could not be detected. However, taken together with the Western-Blot results presented below mAb 1A4 and 1F6 were excluded from further experiments.

| Antibody | Ig Subtype | Detection limit (ng ml^-1^) | Antibody blocking by soluble antigen |
| --- | --- | --- | --- |
| 1A4 | IgG_2b_ | 40 | n. a. |
| 1F6 | IgG_1_ | 120 | n. a. |

n. a. not analysed


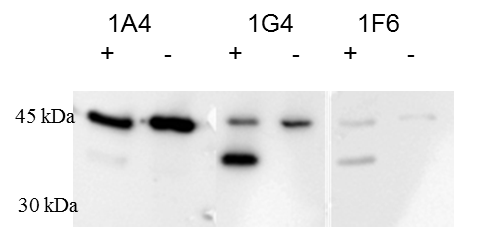


Recombinantly expressed NheA with (+) and without (-) enterokinase digest detected in Western-blot. Membranes were probed with novel mAbs 1A4, 1G4 and 1F6. Upon removal of the thioredoxin tag NheA band should shift down to their correct size at 39 kDa. Results show, that mAb 1A4 preferentially reacts with the thioredoxin tag of rNheA while mAb 1F6 exhibits only a weak affinity for NheA. Thus both antibodies were not suited for further experiments. Only mAb 1G4 showed the desired reactivity and strongly reacted with digested rNheA.
