# Supplementary material for: Antibody Binding Studies Reveal Conformational Flexibility of the Bacillus cereus Non-Hemolytic Enterotoxin (Nhe) A-Component
Source: PLoS One. 2016 Oct 21;11(10):e0165135. doi: 10.1371/journal.pone.0165135 (PMC5074587; doi:10.1371/journal.pone.0165135)
Supplement: S2 Fig — (DOCX) [file pone.0165135.s002.docx]

**S2 Fig** Neutralization of toxic activity from MHI 1507 by mAb 1A8 (▼) and the isotype control (⚫).


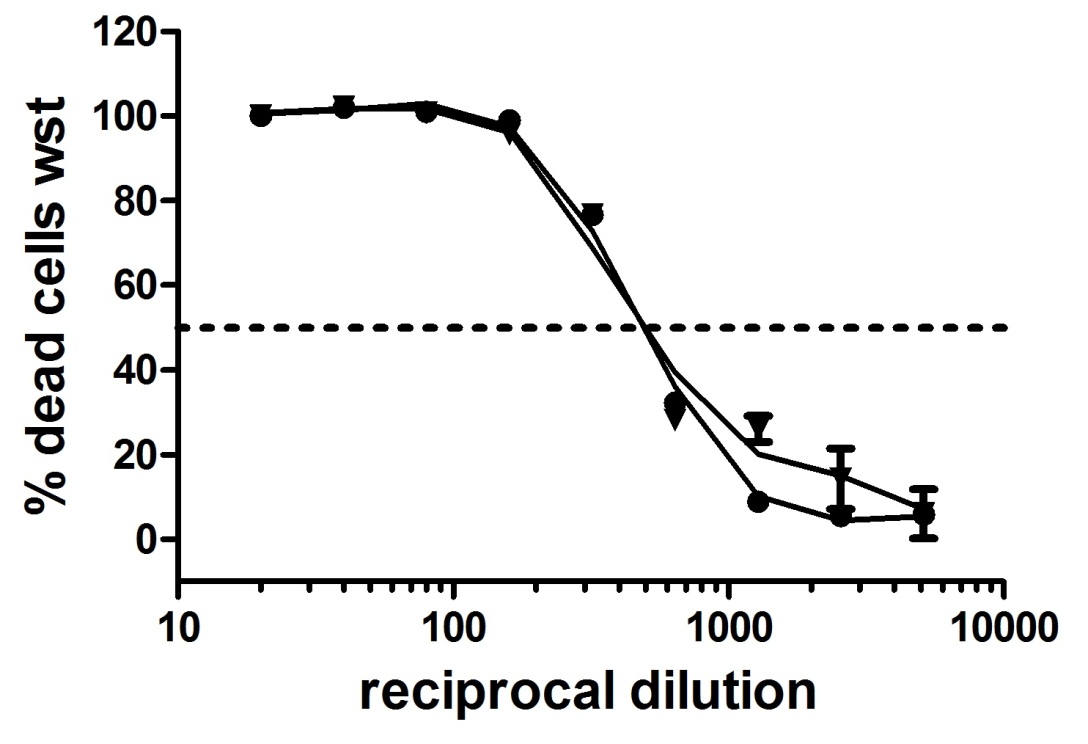


Application of the mAb 1A8 or an unspecific isotype control result in a similar 50 % cytotoxicity titer thus indicating that mAb 1A8 has no neutralizing effects.
