# Supplementary material for: Antibody Binding Studies Reveal Conformational Flexibility of the Bacillus cereus Non-Hemolytic Enterotoxin (Nhe) A-Component
Source: PLoS One. 2016 Oct 21;11(10):e0165135. doi: 10.1371/journal.pone.0165135 (PMC5074587; doi:10.1371/journal.pone.0165135)
Supplement: S3 Fig — (DOCX) [file pone.0165135.s003.docx]

**S3 Fig**

In addition to the classical WST-1 cytotoxicity assay propidiumiodide (PI) influx was assayed on Vero cells. Cells were seeded at a density of 30,000/well 24 h prior to the assay. B. cereus supernatant MHI 1507 was diluted 1:40 in cell culture medium supplemented with PI (10 µg/ml) either with or without 10 µg of mAb 1G4 or 2G11. The mixture was allowed to pre-incubate 30 min before being added to the cells. Untreated cells served as negative control. Measurement was performed on Victor3 multilable counter (Perkin Elmer, USA). Excitation and emission wavelength were set to 530 and 680 nm respectively.


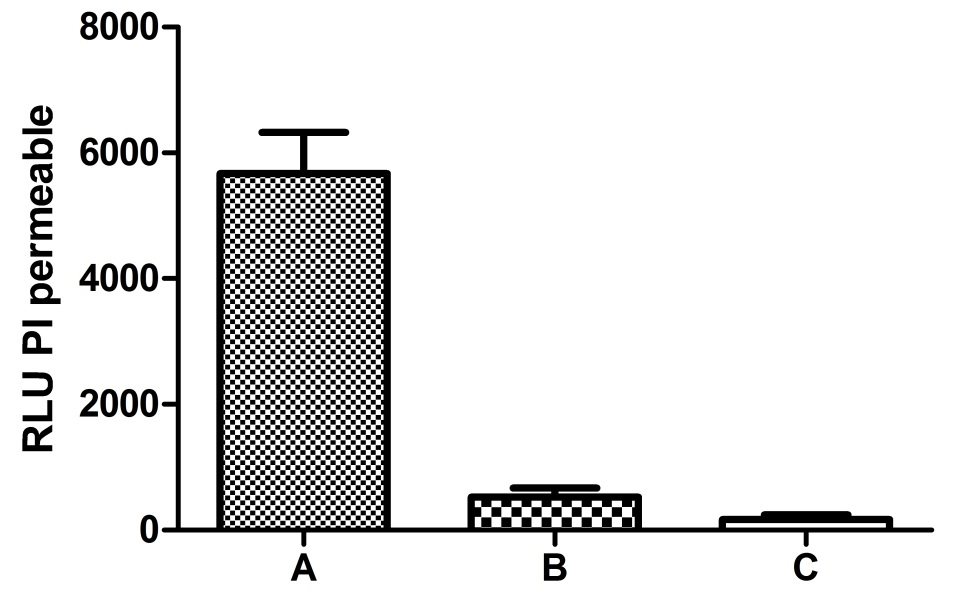


Results of the PI influx assay after subtraction of background fluorescence. Fluorescence intensities are depicted as relative light units (RLU) indicating PI permeability caused by pore-formation. (A) MHI 1507 without neutralization; (B) MHI 1507 neutralized by mAb 2G11; (C) MHI 1507 neutralized by mAb 1G4. Both antibodies are able to prevent pore-formation. Bars and error bars represent means and SD of quadruplicate measurements.
