# Supplementary material for: Antibody Binding Studies Reveal Conformational Flexibility of the Bacillus cereus Non-Hemolytic Enterotoxin (Nhe) A-Component
Source: PLoS One. 2016 Oct 21;11(10):e0165135. doi: 10.1371/journal.pone.0165135 (PMC5074587; doi:10.1371/journal.pone.0165135)
Supplement: S4 Fig — (DOCX) [file pone.0165135.s004.docx]

**S4 Fig**  Depiction of additional controls in flow cytometry


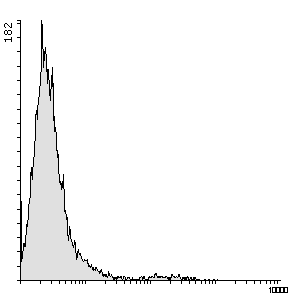


**Fluorescence channel 1**


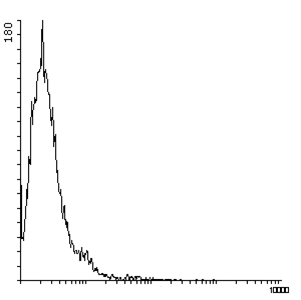


**Fluorescence channel 1**

**A**

**B**

No specific fluorescence is detectable after incubation of Vero cells with rNheA alone (A) or in combination with rNheC (B). The results clearly underline that NheA is only capable to bind to the target cells in the presence of NheB.
