# Supplementary material for: Antibody Binding Studies Reveal Conformational Flexibility of the Bacillus cereus Non-Hemolytic Enterotoxin (Nhe) A-Component
Source: PLoS One. 2016 Oct 21;11(10):e0165135. doi: 10.1371/journal.pone.0165135 (PMC5074587; doi:10.1371/journal.pone.0165135)
Supplement: S1 Table — (DOCX) [file pone.0165135.s005.docx]

**S1 Table**

Nhe expression profiles of *B. cereus* strains relevant for this study.

| Strain no. | NheA | NheB | NheC | cytotoxicity |
| --- | --- | --- | --- | --- |
| MHI 241 (NVH0075/95) Nhe reference strain | + | + | + | +++* |
| DSM 31(ATCC 14579) type strain | + | + | + | +++* |
| MHI 1761 (natural mutant) | -^#^ | + | + | - |

^#^ no NheA expression due to a preliminary stop codon

* cytotoxicity titer >1:400
